# Making and disposing of life’s ‘starting materials’: A focus group study of attitudes concerning reproductive scarcity and abundance in *in vitro* gametogenesis

**DOI:** 10.1016/j.socscimed.2025.118146

**Published:** 2025-05-06

**Authors:** Robbin Jeffries Hein, Anne Le Goff, Hannah L. Landecker

**Affiliations:** a The Institute for Society and Genetics, University of California, Los Angeles, CA, 90095, USA; b SupBiotech, L’école des ingénieurs en biotechnologies, 66, rue Guy Môquet, Villejuif, 94800, France; c Department of Sociology, University of California Los Angeles, Los Angeles, CA, 90095, USA; d Center for Reproductive Science, Health, and Education, University of California, Los Angeles, CA, 90095, USA; e Paris Institute for Advanced Studies, 17 quai d’Anjou, 75014 Paris, France

## Abstract

This paper explores stakeholder attitudes toward gametes and embryos in the context of in vitro gametogenesis (IVG), a new stem cell technology whose future clinical application would entail the production of eggs and sperm in vitro. A key concern raised by the prospect of making gametes from originally non-reproductive body cells has been its potential exacerbation of the issue of “surplus” cryopreserved embryos through the production of unprecedented numbers, with an allied devaluation of human reproductive materials. However, this concern has not been empirically investigated. In this study, focus groups composed of individuals representative of the constituency most likely to be impacted by IVG were asked to respond to scenarios in which the relative abundance and scarcity of gametes and embryos were changed by these new procedures. Respondents who had experienced involuntary childlessness and/or previously accessed in vitro fertilization (IVF) technology drew on these experiences to reason their way through future scenarios in which cells could be more fluidly exchanged over the somatic-reproductive boundary. Unfettered abundance was not found to be a key issue for these respondents. Rather, concerns focused on questions of technological control over outcomes in ARTs, cultural scripts about the preciousness of eggs moderated by concordance between the gender or the donor and the sex of the gamete, and in vitro gametes and embryos as embodiments of the often painful and costly process of attaining them.

## 1. Introduction

Since the fall of *Roe* in 2022, in vitro fertilization (IVF) has received increased political attention due to shifting definitions of personhood and litigation concerning the fate of surplus embryos (Klock and Lindheim, 2022). This was most recently exemplified by the 2024 Alabama Supreme Court ruling that defined IVF embryos as “extrauterine children,” resulting in a temporary halt to all IVF procedures in the state (LePage v. The Center for Reproductive Medicine, 2024). Alongside these legal developments, increased utilization of IVF, advances in cryopreservation, and lax and “highly varied” industry regulations in the United States have led to a perceived crisis in the number of stored embryos (Cromer, 2023, p. 51). Estimates suggest 1.5 million supernumerary embryos are held in storage (Letterie, 2022), posing a challenge for IVF patients struggling with disposition decisions and clinics that must balance patient rights against the ethical, legal, and financial costs of any “unclaimed” embryos (ASRM, 2021, p. 47; Christianson et al., 2020; Goedeke et al., 2017; Lyerly et al., 2010).

**Fig. 1.**
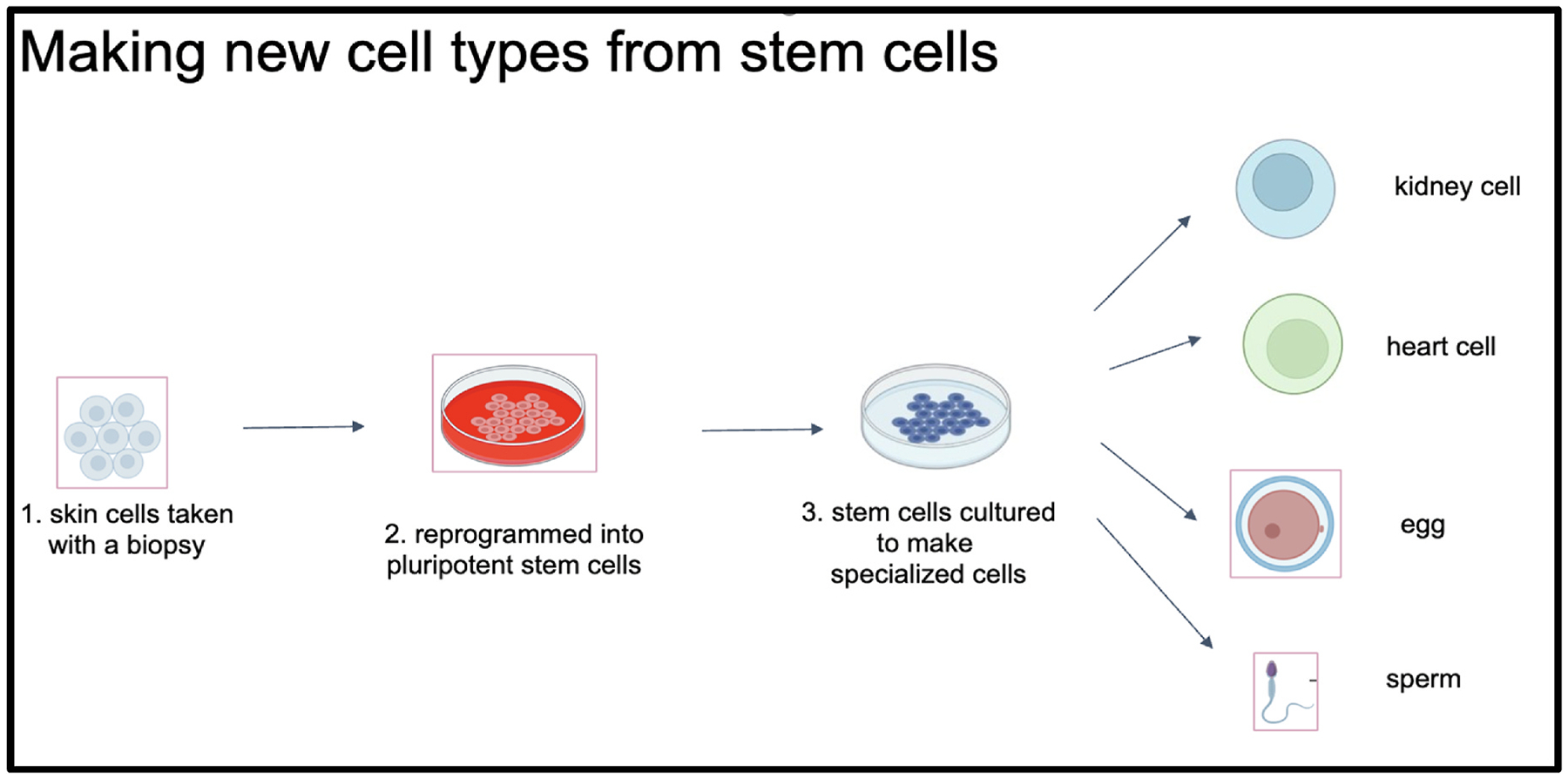
Illustration of IVG presented to study participants This figure illustrates the process of IVG in which somatic cells, for example skin cells, are biopsied from an individual. Through the IVG protocol, these cells are “reprogrammed” to gain function as pluripotent stem cells. From there, researchers further culture the stem cells to become eggs or sperm, and a similar process under different culture conditions could give rise to other cell types.

**Fig. 2.**
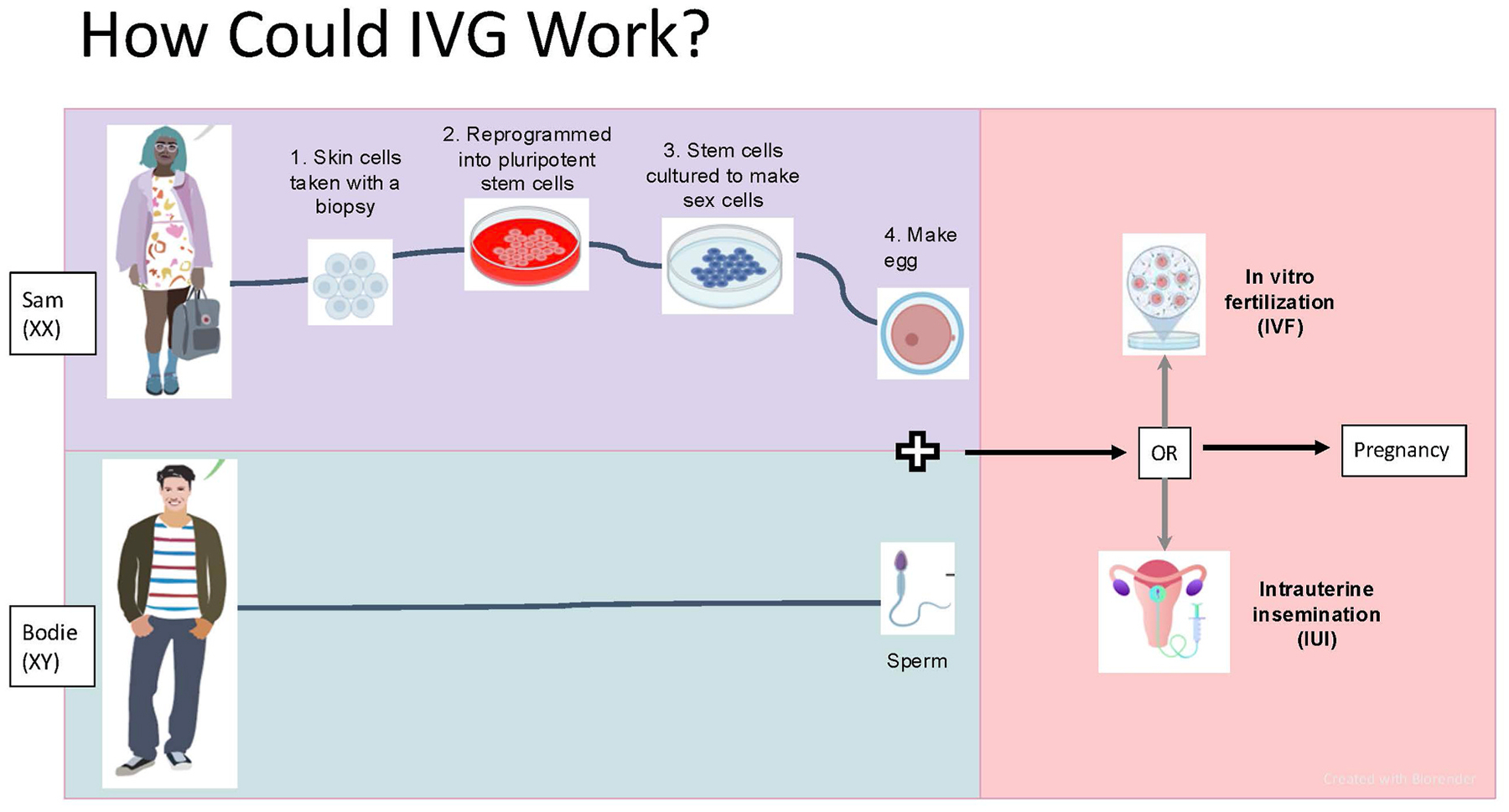
Illustration of how a heterosexual couple might use IVG presented to study participants This figure outlines the hypothetical process for a heterosexual couple using in vitro gametogenesis (IVG) to create viable eggs from Sam’s cells. First, Sam undergoes a skin biopsy to obtain somatic cells. Second, these cells are reprogrammed to a pluripotent state. Third, cells are differentiated into eggs. Once a viable egg is created, it can be fertilized with Bodie’s sperm using IVF protocols. The viable embryos can then be transferred into Sam’s uterus, aiming for pregnancy.

Today, developments in stem cell technology have the potentially to exacerbate these issues. Specifically, in vitro gametogenesis (IVG) seeks to produce gametes (eggs and sperm) by reprogramming somatic cells biopsied from an individual. Cultured in vitro and returned to a pluripotent undifferentiated state, these cells are then induced to become gametes through a series of molecular cues provided by researchers (Chen et al., 2017; Saitou and Hayashi, 2021). In short, scientists reprogram adult, differentiated somatic cells such as skin cells to become eggs or sperm in the lab (see Fig. 1). IVG builds on the capacity for cellular fungibility implicit in stem cell techniques, which de-differentiates and re-differentiates cells fluidly across cell types (Franklin, 2022; Kraft and Rubin, 2016). Proof of the concept has thus far only been achieved in a mouse model (Hayashi et al., 2011; Hikabe et al., 2016), but an explicit goal of researchers is to treat human infertility (Centers for Disease Control and Prevention, 2022; Centers for Disease Control and Prevention, 2021). Should IVG become clinically available, it could enable individuals to become parents to genetically related children regardless of age, sexual orientation, or an underlying medical or physiological condition that impacts gamete availability and/or viability (See Fig. 2).

The “transformative” potential of IVG has prompted calls for *ante hoc* discussions of its ethical, legal, and social implications (Cohen et al., 2017, p. 1–2; Adashi et al., 2019; Clark et al., 2021; Greely, 2016). In 2023, the National Academies of Sciences, Engineering, and Medicine (NASEM) convened experts to engage with IVG’s far-reaching implications. Key concerns included safety, cost, accessibility, eugenics, implications for non-normative family building, social and legal challenges — and the potential for an unfettered production of reproductive materials (NASEM, 2023). In the broader bioethics literature, the phrase “embryo farming” has been introduced to highlight how the human ova supply currently acts as a bottleneck to the number of embryos produced, a constraint that would be lifted if somatic rather than reproductive cells were used to generate gametes (Cohen et al., 2017, p. 2). According to this view, IVG could enable embryo production “on a scale currently unimagined, which might exacerbate concerns about the devaluation of human life” (ibid). Thus, the hypothetical problem of “‘having millions and millions of embryos sitting around in storage’ without clear ways to handle them” might compound complex decision-making about regulating ARTs in post-*Dobbs* America, given there is no national agency that regulates IVF such as the UK’s Human Fertilisation and Embryology Authority (Cattapan quoted in NASEM, 2023, p. 59).

Where the first scientific visualization of embryos in the early twentieth century opened out a role for embryos as “icons of life,” IVG enters a discursive field already overcrowded with conflicting representations of embryos as everything from “reproductive remainders” to persons (Morgan, 2009, p.189; Cromer, 2023, p. 1; see also Morgan, 2002; Crockin, 2005). The potential entry of IVG into these newly invigorated “embryo wars” in the United States is producing a distinctly anxious vision of reproductive abundance. Indeed, such concerns are amplified in popular news accounts of IVG as “mass-producing sperm and eggs in the laboratory” that invoke the “mind-boggling” potential for IVG to produce an “almost limitless supply of eggs made from a scrap of skin” (Devlin, 2025, para. 1; Lewin, 2017, para. 3). Consequently, according to such accounts, “[h]uge numbers of embryos could be created easily and painlessly” in the laboratory, dehumanizing reproduction (Ball, 2018, para. 2; see also Hamzelou, 2022; Regalado, 2017; Sample, 2017; Stein, 2023).

In this paper, we focus on the anticipation of unprecedented abundance as an important element of social appraisals of this emerging technology. As the widespread language of mass production and limitlessness implies, projections of an “inexhaustible” production of “stem cell-derived human gametes and, by extension, preimplantation embryos” carry with them the threat of dehumanization of reproductive materials (Adashi et al., 2019, pgs. 165–166). Such figurations and their unstated assumptions of the relationship between abundance and social worth should be taken seriously, not just because they augment dystopian discourses of reproduction, but because they have the potential to shape new configurations of embryo protection in the legislative landscape.

Importantly, these ideas are being generated by journalists and bioethicists, not the very people who might use IVG were it to become clinically available. IVG’s potential to re-shape reproduction underscores the importance of engaging with stakeholders who are the most likely to engage directly with the technology and whose perspectives can help inform debates on translational research and clinical applications, as evidenced by a recent call from the International Society of Stem Cell Research for greater public engagement (Sugarman et al., 2023). Here, we test presumptions about IVG and its potential to generate gamete and embryo abundance by engaging stakeholders with lived experiences of infertility and/or assisted reproduction. We ask: If it is possible to interchange cells between the somatic and reproductive divide in unprecedented ways, should we expect attitudes to gametes and extracorporeal preimplantation embryos generated from those somatic origins to remain the same? Will people experience the problem of gamete and embryo disposition in precisely the same way, just in larger numbers? To put it most bluntly, if eggs were as common as sperm, how would questions of valuation and disposition shift for stakeholders in the quest to use ARTs?

To answer these questions, we developed an empirical approach to the question of how attitudes towards IVG gametes and resulting IVF embryos might change in such a hypothetical future. To date, literature on disposition decision-making in ARTs has decisively demonstrated the importance of understanding how decisions can shift according to an embryo’s “symbolic representation,” changes in a patient’s life circumstances, and the process of gamete attainment, namely egg retrievals (Bruno et al., 2016, p. 1511; Nachtigall et al., 2005; Parry, 2006). Research on gamete donation has also shown that reproductive context and gender play a crucial role in shaping donors’ views of their gametes in relation to themselves and their recipients (Almeling, 2011; Cromer, 2023). Thus, we hypothesized that altering the process of gamete attainment may impact how IVG beneficiaries view these entities and their attitudes toward disposition decisions.

Drawing on Rayna Rapp’s concept of “moral pioneers,” by which she described women navigating the early days of prenatal genetic testing in light of chromosomal abnormalities, we invited “moral philosophers of ‘the private’” in matters of gamete and embryo generation and disposition to participate in *ante hoc* discussions about IVG with “speculative openness” (Rapp, 1999, p. 3; Meskus and Tikka, 2022, p. 4). Using focus groups and interviews, we explored hypothetical IVG scenarios with people who have experienced infertility and ARTs. Participants discussed how gametes and embryos produced via IVG could challenge current assumptions about these entities and how a change in their relative scarcity might influence attitudes toward them. The results reported below provide empirical insight into participants’ logics in light of IVG’s hypothetical disruption to existing norms and practices. Such anticipatory exercises can offer a “point of orientation for action” in setting the terms of further debate (Oomen et al., 2022, p. 255). IVG, a technology of the not quite yet, will continue to stir controversy about controlling potential dangers of future reproductive possibilities exactly because it departs in important ways from precedents available in natural or social history. Our results obviously do not prove that attitudes and ethical stances will be affected in exactly these ways, but they do constitute an anticipatory exercise in considering how IVG might change not just the number of embryos produced through ARTs but the frameworks through which they are perceived.

This paper proceeds by briefly setting the study in the context of extensive literature on embryo disposition and biovalue, followed by a description of our empirical approach and methods. We then turn to the results. These suggest that alarmed speculation about an unconstrained oversupply of gametes and embryos and an attendant devaluation of their worth or value is unwarranted. Instead, study participants offered several modes of speculation that are important additions to any conversation about anticipating the social and ethical impacts of IVG and other new reproductive technologies. First, our interlocutors articulated hopes that IVG could provide *greater* control over the gamete attainment process, particularly in contrast to current egg retrievals, leading to fewer gametes, not more. Their responses centered on how the current relationship between high uncertainty and low control means acquiescence to pain and expense to achieve hoped-for reproductive outcomes. The future of excess and disposition issues was thus understood as dependent on whether the current calculus amongst these factors could shift with IVG.

Second, contrary to concerns that an unlimited supply of IVG-generated gametes might lead to a devaluation of both gametes and resulting IVF-generated embryos, we saw little grounds for anticipating such a future. Quite the opposite sense emerged. In general, interlocutors hewed to relatively obdurate cultural scripts regarding sperm and eggs even while reasoning through the changes that would come with IVG. Moreover, their thinking about valuation in the IVG context emphasized reproductive materials as embodiments of processes tied to identity in various ways, yielding important insights into the attribution of value to biological entities both now and in the future. Third and relatedly, we found participants reinscribed the somatic-reproductive divide even in pondering IVG-derived materials sourced in somatic tissues. This reinscription was particularly evident in discussions of gametes and embryos donated to research. These findings show that stakeholders with lived experience of a previous generation of reproductive biotechnologies have distinctive and highly original insights to offer as part of an empirically deliberate mode of anticipation of new technologies. How we think about valuation and devaluation is important because it will shape not just moral debates about IVG but the legal landscape of embryo generation and disposition in reproduction.

## 2. Background

While IVG is a new approach to infertility, it enters a well-established landscape of social studies of assisted reproductive technologies and the question of what to do with surplus embryos. IVF patients are routinely asked to select a disposition option for surplus embryos, including storage for future use, donation to research or others, and disposal (Raz et al., 2021). Disposition decisions are widely described as “distressing and emotionally loaded” (de Lacey, 2013, p. 31). Patients have characterized ART-derived embryos along a spectrum from “virtual children” (de Lacey, 2005) to “embie-babies” (Becker, 2000), a “potential person” (Bruno et al., 2016; Delaunay et al., 2023), and future “kin” (Kvernflaten et al., 2022). More dispassionate framings have also been reported. These include descriptions of embryos as a “bunch of cells” or “base material” (Fuscaldo et al., 2007, p. 3136; de Lacey, 2007, p. 1755). When notions of the “moral status” of embryos do arise, they are situated on a continuum from “full moral status comparable with a person [to] no moral status comparable with a thing” (Provoost et al., 2009, p. 899). Other disposition research stresses the impacts of structural and life course factors, including gender and reproductive norms, legal regulations, financial security, relationship status, age of female patients, and timing of family completion on patient perspectives (Lyerly et al., 2010; Samorinha et al., 2014).

Additionally, research emphasizing the impact of ART’s intensive technical procedures on people’s views is highly relevant as we consider the addition of new laboratory-mediated techniques. For example, studies have found that some IVF patients attribute “high instrumental value” (Provoost et al., 2009, p. 900) to embryos based on the “significant time, energy, and financial investment” made in attaining them (Goedeke et al., 2017, p. 1535). De Lacey (2007) illustrates this point in her analysis of decision-making in which women participants characterized embryos as “hard won” as a result of ‘what we went through’ or the ‘trouble it took’ to ‘get’ or ‘make’ embryos in the first place” (p. 1755). As we illustrate below, our findings also indicate that processes of gamete procurement — which are physically grueling, expensive, and emotionally complex, or in many cases altogether unachievable — shape attitudes and inform reproductive decision-making (Waldby and Carroll, 2011). Thus, the ideas people have of gametes and embryos are not derived exclusively from abstract moral principles about the origin of life or ontological discussions about personhood.

While our study’s empirical materials lie in the domain of studies of public and patient attitudes, we also situate individual perspectives in the larger anticipatory currents of biotechnology’s reconfiguration of “bodies and their parts” (Adams et al., 2009, p. 252; Cooper, 2011). Gametes and embryos circulate today within a larger context of a “biomedical model of reproduction,” an era characterized by markets for cells and tissues as sources of promissory potential (Thompson, 2005, p. 247; Franklin, 2006; Helmrich & Labruto, 2018; Waldby, 2002, 2019). Studies of tissue economies, including stem cells derived from donated embryos, illustrate how these entities begin to function as promissory capital because of their potential role in creating new therapeutic medicines (Waldby and Mitchell, 2006). In turn, cells, tissues, and organs can generate new forms of biovalue and ethical considerations in biomedicine, a sequence of events already visible in the simultaneously figurative and literal investments in IVG as the next generation of ARTs as promissory technologies. For example, gamete commodification could be accelerated, turning reproduction into a scalable commercial industry that further generates novel biologics and therapies, new forms of capital, and legal and regulatory frameworks outlining unique concerns around the patentability of IVG gametes as proprietary products (Cyranoski et al., 2023).

This study builds on a small but growing international set of social science literature on IVG in which stakeholder attitudes toward stem cell-based interventions for reproductive care have been surveyed among gynecologists, infertility patients, and the general public in the Netherlands (Hendriks et al., 2017, 2018), the general public in Belgium regarding IVG’s future potential applications (Mertes et al., 2022), and acceptance of IVG derived gametes and embryos in Japan (Sawai et al., 2021). Qualitative research methods have also been utilized to study IVG researchers, ethicists, and patient representatives in the Netherlands (Cutas et al., 2014), men and couples experiencing male-based infertility (Hendriks et al., 2014, 2016), and most recently, prospective users’ hopes and concerns around IVG in the United States (Le Goff et al., 2024). Bridging between studies of embryo disposition within existing ART practices and this small body of work on attitudes toward a potential ART, this study’s specific focus on anticipating future shifts in abundance and scarcity of reproductive materials with IVG offers novel empirical insight into both current and possible events.

## 3. Methodology

### 3.1. Participants and recruitment

This study was approved by UCLA’s Institutional Review Board (Protocol #21–001281) and followed the Transgender Research Informed Consent disclosure policy. Our findings are part of a larger qualitative study conducted in 2023 on the ethical, legal, and social implications of IVG and its potential clinical use.

We consulted with the UCLA Community Engagement and Research Program and leaders from fertility support services for LGBTQ+ and BIPOC communities. Based on their feedback, we developed recruitment flyers and distributed them to reproductive health organizations in Los Angeles] and online social media accounts related to reproduction. The recruitment flyers included a QR code that applicants used to answer an eligibility survey. The short survey included questions about infertility and demographic data, allowing us to create a diverse pool of applicants across gender, sexual orientation, race and ethnicity, income, and geographic location within the US. More than 450 applicants responded to our recruitment flyers.

We recruited participants who had experienced involuntary childlessness for medical or social reasons. Our sample included cisgender heterosexual individuals and LGBTQ + individuals who had experienced infertility or involuntary childlessness. Analytically, we focused on these groups for two reasons: they had prior experience with rare or absent gametes, and many of them had experience with IVF embryo disposition decisions. As such, participants were uniquely positioned to consider how and why a shift to gamete and embryo abundance might matter to their thinking. All participants were 18 or older, spoke English, and lived in the United States. We provided consent forms to participants and financial compensation for their time. Table 1 reflects the self-reported demographic characteristics and gender and sexual identity of all participants.

We conducted 10–15-min screening interviews with respondents via Zoom. The initial consultation helped us ensure that participants understood the scope of the study and confirmed their eligibility, particularly in terms of lived experiences of infertility and access to the Internet for online participation. We also utilized snowball sampling within our participant pool to redress an initial underrepresentation of gay and heterosexual men, BIPOC, and gender non-conforming individuals. A majority of participants had a relatively high household income in accordance with the high financial barrier for people using or considering ART in the US, who represented our target sample. Following the screening process, we recruited individuals to participate in focus groups.

Focus groups provide a valuable method for uncovering and detailing various perspectives, including perspectives on new and potentially disruptive technologies such as IVG. Follow-up semi-structured interviews with a sample of participants allowed us to explore further how people make sense of new reproductive technologies and how that might impact their views on ARTs. Qualitative methods provide rich detail, but they may introduce bias from participants and researchers; they also lack generalizability to larger populations because of their small size and non-random selection (Denzin and Lincoln, 2005). Nonetheless, insights gained from qualitative research can help develop variables and hypotheses that can then be tested systematically via quantitative methods.

### 3.2. Data collection

Between March and August 2023, our research team conducted eleven online focus groups moderated by two team members. Eighty interlocutors participated in 11 90-min focus groups of 3–7 participants and/or one-on-one interviews. After introductions, moderators gave a 15 min PowerPoint presentation describing IVG in lay terms, during which participants were encouraged to ask questions and share their general views and impressions. Next, participants considered the stages of IVG research needed to advance it to a clinical stage. Participants then discussed hypothetical scenarios of infertile people and how IVG might be used in different clinical contexts, as well as the broader social implications of IVG including accessibility, financial costs, and implications for LGTBQ + minorities (See Table 2). Participants highlighted themes of health and safety, including quality of gametes, gene editing, potential misuse, accessibility, expansion of ARTs to non-normative families, improved reproductive success, and the alleviation of emotional and physical pain resulting from invasive procedures (Le Goff et al., 2024).

Study participants were invited to apply for a semi-structured interview with one team member. During this stage, we again sought a socially diverse group. We drew on themes identified in the focus groups to structure our interview guide but allowed participants to share topics of interest as they unfolded during the interview. This helped build additional rapport and facilitated a conversation that enabled the interviewer and interviewee to share complex experiences that shaped their views of IVG. We conducted 25 semi-structured interviews between May and September 2023, ranging from 60 to 90 min each. All interactions were conducted using Zoom video conferencing with automatic transcription. In total, we collected 43 hours of recorded participation. Team members de-identified and edited the transcripts for accuracy.

### 3.3. Data analysis

Data were analyzed using a grounded theory approach (Charmaz, 2006; Glaser and Strauss, 1967) to identify central themes across the data, highlight variations, and develop a descriptive analysis of how participants created meaning around IVG gametes and embryos while situating their experiences of infertility and ARTs. Two team members independently read and coded the transcripts, generated codes, resolved differences, generated themes based on the codes, and collaboratively created a refined codebook using Dedoose software to organize data.

## 4. Results

Our findings below are organized into three sections. First, we show how infertility treatment experiences led to an emphasis among our interlocutors on control with IVG. They saw IVG as a better alternative to current technologies if it could improve processes of gamete attainment and reduce excess embryos by providing more precise control of the process. Second, we examine how thinking through potential shifts in gamete scarcity to abundance affected people’s perceptions of these reproductive materials, with gender identity an important factor in talking through how IVG-generated gametes and embryos could be valued. Third, we analyze differing reactions to the hypothetical donation of IVG-derived cell types for research, an important question given the dependence of any move toward clinical applications in humans on research with human-derived materials.

### 4.1. In vitro gametogenesis: Controlling for reproductive abundance

In the focus group exchange below, cisgender men and women highlighted IVG’s potential to generate viable gametes and avoid painful and emotionally difficult ART procedures.

Sammi: We were never able to retrieve eggs, ever … [T]hey all stalled out, and so that’s why both IVFs were canceled … So it would be great to be able to like [do this], and also not to take those medications, because it was horrible.

Veronica: I’m not even able to do IVF. I was told right away it’s not even an option for me because my AMH level is, like, undetectable. I can get pregnant on my own, but I can’t keep it. So, it’s an egg quality issue, so [IVG] seems really exciting like, ‘Well, now there’s finally an option.’

Tom: My male factor is unexplained. It’s idiopathic, like we don’t have it [sperm], and we don’t know why. After lots of poking and prodding, [there’s] no explanation. And so, yeah, having an avenue that would give results without, you know, having to do biopsies of places that would rather not be biopsied would be, you know, attractive.

Significantly, these interlocutors resisted the idea of IVG generating a problematic excess of gametes because they were speaking from the experience of extreme or total gamete scarcity in their own or their partner’s body. The capacity for abundance appeared *as a solution*, not a problem. Participants also considered whether IVG technology might be better than current ARTs in exerting greater control over the number of gametes acquired.

Tom reflected on this possibility:

[I]f this technique does take off, [it] … opens up the [question], ‘What happens to the extra embryos?’ mainly if you’re saying, ‘Okay, let’s do lots.’ But I wonder why you would necessarily need to do lots if this technique is probably fairly easy. The reason we produce so many embryos through IVF is that it is such a difficult and expensive process to do one retrieval. You don’t want to just pluck one or two eggs. You want to pluck as many as you can get.

He continued to explain:

[P]art of the reason why you’re doing embryos is that they freeze better than the eggs or the sperm, individually. With this technique, that doesn’t matter anymore. I would think that you’d probably be producing less embryos just in general.

Jason, a cisgender heterosexual man, described IVG technology as “like another tool to use.” Yet he too wondered whether “you [can] control how many eggs are created, or how many sperm are created through the process they use.” Paul, a cisgender heterosexual man, reiterated the question of control, placing his response in the context of his and his wife’s infertility journey and a changing legal landscape in their home state:

We had excess eggs in our last cycle, and we had to make a tough decision being in Texas, where there’ve been some political issues surrounding the discarding of fertilized eggs [embryos]. And so we’d already decided that we were not planning on moving forward with another [cycle]. And so I think there–it’s gonna be a big portion of the population who will have a big concern with anything to do with new technologies involving excess eggs. I know, I think, that would be a critical, a critical point in this technology.

These participants highlighted not only their individual feelings about IVG but wondered how other people would respond to changes in gamete abundance or previous restrictions on generating embryos. Sarah, a cisgender lesbian woman, put it this way:

I’m curious as to how many eggs, sperm, and potential embryos would be produced in any given round of IVG. I personally don’t have concerns when it comes to, when you phrase it ‘disposal,’ it sounds more like a negative connotation, but ‘not using all of the cells or the embryos,’ I suppose, is the way we can say it. I almost imagine maybe there would be a tipping point … [I]f somebody said there were 22 viable embryos, will that change my opinion of whether or not discarding or disposing of those would be of any more ethical concern than the fact that we had two [embryos] that were viable that didn’t get used?.. I don’t have a personal concern, but I could see where there might be if there was almost an excess of viable embryos and cells created from the process.

Control comes up again here implicitly, in the sense that Sarah speculates on a “tipping point” that the purveyors or users of this technology should be careful not to cross — although she is uncertain as to the exact location of such an ethical line beyond which there are too many to bear.

Anusha, a cisgender heterosexual woman, described IVG as “a slam dunk” and remarked that she would “100 % rather prefer to do something like that than have to undergo an egg retrieval.” However, she also stated:

[O]ne thing that would kind of concern me [is] maybe you could actually control (since it seems like this is a process that has a lot of control kind of baked into it)– the number of potential eggs that would result because one of the things that I personally struggled with [was] my embryos, which are not the same thing as eggs, I know that. It’s just that, if we decide not to [have] another child, I know that I can discard them, I can donate them, I can give them, you know, to science, all of that. But it’s still very personal, and it’s a very challenging decision to make. And it’s one that even the process it takes to get there, it feels like losing a little bit of yourself, right? And I wonder, if you have the IVG process instead, are you at the same risk of having these banked eggs or embryos, and then you have to deal with this issue of, ‘What do I do with what I have left?’

For Anusha, the *process* of IVF was such that the embryo embodied what “it takes to get there,” and therefore any disposition “feels like losing a bit of yourself.” Would IVG, by changing the process, shift that sense — or not? *Would* it confer more control over the number of eggs and embryos made in any given effort to have a biological child? As with many respondents, Anusha could reason by extrapolating from her lived experience into a hypothetical future of changed constraints. Yet there are limits on speculation; the exercise allowed her to clearly articulate the question while remaining unsure of how she would really feel about an embryo that embodied an IVG process instead of a conventional egg-retrieval process.

### 4.2. Conceptualizing eggs and sperm in the IVG context

Here, we turn to participants’ views of eggs and sperm in the IVG context. Because IVG can theoretically produce viable eggs in a large quantity, would this changed parameter redefine eggs as less precious? Would IVG shift understandings of sperm? We also explore participants’ responses to the production of non-matching gametes, i.e., gametes that do not match one’s sex chromosomes, to explore how this may also impact gamete valuation.

Helen, a heterosexual woman diagnosed with infertility, offered the following interpretation of gametes in terms of their equal availability:

I think, right now, I do [see them differently] because right now, in our world, there are way more sperm than eggs. But, if we are in a research facility, and we can create the same number, then I don’t think I would value them differently. I don’t think I would see them very differently.

Even though Helen was able to envision egg abundance, hypothetically allowing her to value gametes equally in the IVG context, she still had a difficult time imagining it:

It’s really hard for me to step out of my 36 years of learning. We are born with just a few eggs, and eggs are, you know, so few, and there are millions of sperm. And so that’s hard. But I understand, logically, that if you’re in a lab and you make 1 [egg] and 1 [sperm], 2 and 2, 3 and 3, I don’t know, I see them [as] the same.

By contrast, Stacey, a queer woman living with infertility, explained why eggs might continue to be assigned greater cultural significance, even if they were equally available:

A lot of people, when they think about when conception starts, it’s much more centered around the egg. So I could see them being like, ‘Oh, well, sperm’s fine, but [eggs]? That’s too far.’

Notably, Sarah, a cisgender lesbian woman, provided an insight into how IVG eggs might *increase* in value:

I think the egg would probably be valued more just because the cost and the process of extracting eggs are much more intense than the process of extracting sperm … I think it’s almost ironic that the egg would be … more valued, [but] it makes sense just economically [that] it would be valued more—to not have to do hormone treatments and the egg extraction process. But I also think because it would basically be like, well, the woman wouldn’t be as necessary.

Thus, Sarah connects gamete valuation to experiences of the procurement process, arguing that because IVG eggs would replace egg retrievals, they may become more valuable, not less. She also contends that valuing IVG itself would come from its potential to replace the ovulating person as the necessary origin of eggs. Whereas Stacey honed in on cultural understandings of gametes that shaped her account of them as differently valued, Sarah focused more on the potential devaluation of women, not eggs.

Among cisgender, heterosexual men who lacked functional sperm, the prospect of IVG-derived sperm was “huge” for some (Kevin). In part, this was because treatment for male infertility is difficult—Tom quipped that “andrologists are unicorns,” a reflection of gendered disparities in reproductive health and science (Almeling and Waggoner, 2013; Almeling, 2020). In their experience, urologists tend to focus on male “performance,” and, at the same time, fertility medications prescribed to women are “not approved for male use” (Tom, Stacey). Another participant, Alan, calculated that undergoing surgery to have a “5 % chance of finding sperm” was not a “good risk.” Like the women described above, these participants focused on practical and embodied aspects of their experiences, such as pain, costs, and the effects of IVF treatment on their female partners. For men, IVG sperm also represented a “hope technology” (Franklin, 2013, 2022; see also Becker, 2000; Inhorn, 2020).

A second way we sought to understand possible differences between eggs and sperm was through the scenario of non-matching gametes, meaning the production of gametes that do not correspond to one’s sex chromosomes. This possibility, for which a proof of concept was given in a mouse model, could change the calculus of biological reproduction for LGBTQ + individuals (Murakami et al., 2023).

Jules, a cisgender lesbian woman, reflected on the possibility of having IVG-generated sperm created from her somatic cells:

I think of [eggs] as a more precious commodity, which is why, I think, and I think maybe as a female, I know that my contribution to life is an egg, and I guess it would be kind of hard for me to wrap my head around, like, me producing sperm … I think that it’s probably why I’m like, ‘My sperm’? I don’t know … [Y]ou know, it’s kinda like losing something or being given something you didn’t even really know you had.

Jules connects her feelings about a non-matching gamete - sperm - and its disposal to her embodied experiences of producing eggs and her gender identity, arguing that value depends in part on concordance between the two.

Carrie, a cisgender bisexual woman, provided a similar view to Jules, imagining social reactions to it:

I definitely think society would really look askance at any non-matching gametes from a person that they expect. So a sperm cell from a cisfemale, I think people would definitely be like, ‘What the hell?’

As for the question of excess sperm and their disposition, she jested: “Put ‘em in a gym sock. I don’t care. Yeah, whatever. I mean, worldwide, on a daily basis, a bajillion of those are getting wasted anyway.”

By contrast, from the perspective of queer and trans participants, IVG sperm *were* ascribed unique value precisely because the gamete accorded with one’s gender identity. Kirby, a trans man, illustrated the importance of this alignment for understanding how gametes become valued. In the context of a discussion around pregnancy and hormone replacement therapy, he explained, “I would never want to carry a child myself; that would freak me out really bad.” Kirby added:

I don’t want the process of harvesting eggs. Like, that’s not for me. I had a period where I was really broke, and I was looking into selling eggs and ultimately came to the decision that this would … [take a] toll on my mental health. I’ve never taken birth control because the thought of putting more estrogen into my body really freaks me out and makes me super uncomfortable as a trans man. So the thought of IVG, where it wouldn’t mean that I would have to stop taking hormones … is a much more appealing option.

A queer woman, Patricia, expressed parallel enthusiasm about IVG sperm in an LGBTQ + context, imagining:

[I]t would be huge, especially for gosh, for everyone, but I can imagine trans folks, how it could be potentially very affirming. … I can imagine that being a very amazing, euphoric process for someone.

These responses, while varied, illustrate *relational* aspects of the conceptualization of gametes to their valuation, including disposition. These include the procurement process for women and men, cultural scripts, and the concordance between gamete embodiment and gender identity. Each of these factors reflects additional aspects for understanding how people view gametes in present ART configurations as well as speculative ones.

### 4.3. Surplus gametes, embryos, and somatic cells in a research context

In this final section, we describe participants’ views on donating surplus IVG gametes and resulting IVF embryos to scientific research. Clinical validation of IVG gametes will only be achieved by an extensive research process that will inevitably involve the creation and analysis of human embryos that, in turn, will necessarily be destroyed within the research process. It is therefore important to gauge how people who might be in the position to donate these materials to research feel about it. We also asked participants to consider a scenario in which they donated somatic cells that would be reprogrammed to become either a somatic cell type, such as a liver, or a gamete to explore perceived differences between cell types. Tom took a pragmatic approach to research donation, explaining:

The natural process ends up discarding an incredible number of embryos as well, and between not implanting and early miscarriages, it’s part of the natural process. I don’t have a whole lot of difficulty with it being something that’s done. And the results should, hopefully, have far greater benefits in the long term.

The idea that embryos are lost as “part of the natural process” made it easier for Tom to imagine this scenario and revealed an underlying logic of naturalization. It also suggested a logic of efficiency and productivity in that he would want any surplus mobilized to help advance science rather than be discarded.

Cynthia, a cisgender bisexual woman, framed her response in the context of current IVF practices, noting how routine loss “already” occurs:

Embryo grading is a subjective process done by embryologists. Some IVF labs will discard embryos. This is already happening … As someone who relies on a medication that’s probably created through cell lines that were from abortions way back, my life relies on those… I just don’t see this as research material … only for having children. There might be other offshoots that could help treat diseases.

Like Tom, Cynthia viewed embryo loss as something that routinely occurs via IVF and donating them to scientific efforts as an act of beneficence. In her view, these reproductive materials may give rise to more life in the sense of giving life to people suffering from diseases whose treatments were enabled by using them.

Darrell, a heterosexual man, described his openness to research donation for these very reasons, stating:

I don’t personally see any issues from a moral standpoint of having the extra embryos researched and studied … [T]hat’s something people have been doing for decades, which has led us to the technology that we have today. [I]f there’s anything that me and my wife can do with leftover, excess specimens … and that we have consented to research, if that helps pave the way for greater and better technology so that people don’t have to go through what we’ve gone through, like, I’m all for it, definitely.

As Sarah remarked, there is “the added value of being able to continue to utilize the cells, the embryos, whatever it may be, at different stages to advance the research further.” Anusha also indicated a strong preference, saying:

I would 100 % donate to research … I would not want to just dispose of them, like, just literally have them tossed out into the trash.

While these excerpts illustrate participants’ willingness to donate their excess reproductive materials to science, others were more hesitant. For example, Grace, a heterosexual woman, considered research that may “improve people’s, or lessen people’s suffering” a “valid use” of remaining gametes and embryos. However, she was concerned that “if the subsequent egg was fertilized, that it [the embryo] would not be treated frivolously” and would “want information on how the egg was being used” by researchers. Likewise, Brian, a cisgender gay man, described embryos as “cells” that are “not a human,” yet he also characterized them as “precious, early material for life.” In this context, Brian added that he would want scientists to be “thoughtful about how they’re researching, or using them, and disposing of them.” From Grace and Brian’s perspective, gametes and embryos retained special importance, a position held by, Lucia, a cisgender woman who felt strongly that if embryos were donated, they should be donated to other people wanting children.

Second, we also asked participants to consider a scenario in which they donated somatic cells (again, derived from a skin biopsy) that would be reprogrammed to become either another somatic cell type or a reproductive cell.

While Grace was “down for” donating somatic cells, she also explained:

[It is] a little bit different because I guess sperm and egg cells are like the genesis of life. I guess it would depend on how my skin cells were taken to grow like an egg, like how the egg would be used. Yeah, and I’m not even like a very religious person, but I feel like it is a little bit different than if you were to grow like, a liver. But yeah, I guess like, if the egg were then inseminated and life was created, I would feel kind of responsible for that life, so I would want information on how the egg [embryo] was being used.

Carrie voiced a similar response, stating:

I would definitely need to know what is happening with the [gametes], for sure. Yeah, liver, whatever I don’t care about … I mean, it’s probably fairly obvious–the possibility to potentially create a child from it. I would not want that to happen without me knowing about it, definitely not.

These responses indicate a general willingness among our participants to contribute biological materials to research, but there were caveats. While many remarked that research disposition is something “people have been doing for decades” (Darrell), some interlocutors felt special care should be taken with cells that are made reproductive. This finding itself may not be surprising, but what it implies is. Even though IVG reconfigures the somatic-reproductive boundary and theoretically could make any cell reproductive, thereby changing the meaning of *all* cells, our interlocutors stayed with the divide. That is, cells with a reproductive potential remained different in their thinking and, because of that, were accorded different values.

## 5. Discussion

To our knowledge, this is the first study investigating potential users’ views on IVG’s capacity to generate abundant reproductive starting materials and therefore questions of what to do with “leftover, excess specimens,” as one of our participants termed them (Darrell). Unlike scenarios depicted in popular media and bioethics literature, in which IVG is assumed to lead to a scene of overabundance and devaluation of embryos, several important alternative considerations emerged which we will discuss in turn. Though participants offered a range of perspectives and presented contrasting viewpoints in reaction to one another, several notable patterns emerged across the dataset: (1) the centrality of control to anticipating abundance scenarios, (2) enduring cultural scripts as forces in valuation key to anticipating changed conditions, and (3) reproductive materials as embodiments of “the process to get there,” regardless of the potential somatic provenance of gametes achieved in vitro. These findings demonstrate the importance of engaging with experienced stakeholders to explore the potential research and clinical implications of IVG, as the majority of these ideas have not, to date, been visible parts of the discussion around the technology’s potential impacts on embryo and gamete disposition.

The first pattern concerns control. Having gone through arduous, expensive, and pharmaceuticalized IVF treatments in the name of technological control of reproduction that paradoxically produced a sense of lack of control (risk of ovarian hyperstimulation, number of eggs retrieved, failed cycles), our interlocutors saw controlling these factors as a key feature for discussion. While participants held differing views, they did not see a putative explosion in the problem of excess gametes or embryos as inherent to IVG. Nor did hypothetical excess lead in any obvious way to their devaluation. Instead, participants focused on how IVG might *better* control a procurement process currently characterized (in their experience) by a lack of control. As the existing literature on ARTs illustrates in detail, many aspects of current fertility practices, such as gamete retrieval, surplus disposal, and fertility preservation are deeply constrained by age and the biological condition of reproductive tissues on the one hand, and risks associated with ovarian hyperstimulation and multiple embryo transfers on the other, constraints that IVG would potentially circumvent by starting with somatic tissues (Brown and Patrick, 2018, p 966; Inhorn, 2017, 2020; Wu, 2023).

The ability to work around some of these previously seemingly immutable constraints led our interlocutors to speculate that changing the degree of control—whether it was “quality” control or more certainty about egg production—could lead to *fewer* gametes (Tom; cf. Wahlberg, 2018). Thus, while the age-related issue of having too few eggs to use for IVF was paramount in participants’ interest in IVG, they were not so much hoping for more eggs as “better” eggs and, to a certain extent, sperm, i.e., gametes that would be more likely to lead to a successful pregnancy. Particularly for those who struggled with diagnostic odysseys and repeated IVF failure, IVG seemed to represent a *reduction*, not just of gametes or embryos, but of tissues extracted by biopsy, cycles of hormone injections, invasive tests, and the number of years spent trying to attain gametes. The broader significance of this was illustrated by Paul, who emphasized the restrictive effect of new state laws surrounding surplus embryos on his and his wife’s ability to continue their fertility journey with IVF. As other studies of stakeholder attitudes have found, IVG represented an attractive option for participants compared to conventional ARTs, its processes, and its failures to produce the desired outcome precisely because of its potential to better control existing practices (Tom; Le Goff et al., 2024).

The second theme revolves around the obduracy of specific cultural scripts. Even in a hypothetical scenario of gamete abundance, we found that cultural meanings remained salient as they were “transmuted into bodily process” (Almeling, 2023, p. 767). It might seem paradoxical that the same people can hold fast to deeply entrenched ideas about the value of eggs while also being open to “talking about something that’s completely rewriting how we think about reproduction,” as one participant put it (Brian). Culturally embedded notions around parenthood and gendered expectations for reproductive responsibility can accommodate new understandings of what is technologically and socially possible (Franklin, 2013; Friese et al., 2006; Oudshoorn, 2003). It is precisely because these perspectives are embedded in experience and contextually reasoned that these interlocutors could imagine how ideas of value, preciousness, or sacrosanctity could be *recontextualized* and operate as forces in moderating and regulating novel capacities to manipulate human matter.

The cultural value attributed to eggs, for example, was discussed as a primary shaping force for the conduct, cost, or regulation of egg generation. This meant that should IVG generate an excess of eggs only for them to be disposed of, it might constitute going “too far” for both individuals and society (Sammi). This view of eggs, as described by cisgender women, reflects a notion of womanhood and identity that includes eggs as one’s contribution to “life,” as Jules explained it (Becker, 2000; Thompson, 2005). Notably, trans and queer participants did not attribute a sense of preciousness or scarcity to eggs. As we saw with Kirby and Patricia, the embodiment of a gamete that does not match one’s gender identity can be a source of discomfort that IVG could alleviate. For trans people, it would achieve a concordance between embodied gametes and gender identity (Armuand et al., 2017; Birenbaum-Carmeli et al., 2021).

This finding builds on arguments that attitudes toward gametes are situationally bound. As with cisgender individuals, gametes are valued *when* they accord with gender identity. That is, gametes do not possess inherent meaning or value. Of course, for gay and lesbian couples, producing a non-matching gamete would also allow them to achieve biological parenthood, creating valuation primarily driven by the quest to become parents (Le Goff, 2024). Thus, rubrics for valuation and therefore disposition should be understood as intersectional outcomes worked out as a combination of past experiences, persistent cultural scripts, gender, and a sense of a change in the fundamental “rules” of biology. Rather than seeing new technology as changing people’s values, our participants were more likely to think about how deeply-held beliefs would inflect new technologies.

Finally, we found that the meanings and values ascribed to life’s starting materials are deeply structured by processes of attainment. Drawing together the cultural logic of eggs as precious with the lived experiences of IVF procedures, one participant hypothesized that IVG-derived eggs might become *more valuable* exactly because they would replace egg retrievals. The routine description of egg retrievals and testicular biopsies for azoospermic men as painful suggests that *both* gametes could increase in value not because of the process of IVG itself, but the retrieval process that it allows the avoidance of (Almeling and Wiley, 2017; Hendriks et al., 2014; Wahlberg, 2018). Thus, IVG may produce outcomes that do not necessarily involve overproduction and undervaluation of life’s starting materials (Cohen et al., 2017, p. 1–2).

The process of gamete attainment also structured valuation in our hypothetical scenarios of research donation. Regardless of their provenance, most participants favored donating somatic cells, surplus gametes, and embryos to research. Rather than opting for disposal, our interlocutors invoked language such as creating “greater benefits” for the “greater good” (Darrell; Provoost et al., 2009). Given the emotional, physical, and financial costs invested in gamete procurement, participants did not want to see surplus materials go to waste (de Lacey, 2007; Millbank, 2017). However, it must be noted that exactly because IVG repurposes cells across the somatic-reproductive boundary, some participants were more apprehensive about research donation. For example, if donated somatic cells were subsequently reprogrammed to become reproductive cells, participants expressed a sense of responsibility toward them. In turn, reprogrammed reproductive cells would require a different “kind of care,” suggesting that IVG is unlikely to unsettle current practices of research-based donation (Pfeffer, 2008, p. 2548). Though one might expect that the increased fungibility across cell types would erode the preciousness or sanctity of reproductive materials, interlocutors repeatedly underlined how reproductive materials are conceptualized and valued as embodiments “of the process to get there,” rather than being seen as an intrinsic value set by the provenance of the cell.

## 6. Conclusion

Considering emerging technologies before they are in place is an important exercise in anticipating and informing public and policy discussions as these developments unfold. In a society where ARTs are increasingly used and politicized, it is important to bring diverse stakeholder views to the work of anticipation, including those for whom IVG might be the most impactful. Deeply thoughtful, these considerations of future development in ARTs are as much about the present as they are about the future. They add empirical insight to the literature on the disposition and valuation of human tissues regardless of whether IVG ever reaches clinical application. While necessarily particular to interlocutors highly invested in assisted reproduction, the benefit of engaging people with lived experience of infertility and IVF can be seen in the way the responses detailed here disrupt any easy assumptions about how future new reproductive technologies will unfold as social, not just biotechnological, processes.

## Figures and Tables

**Table 1:** Demographic characteristics of study participants (n = 80).

**Age (years)**	
18–23	1 (1.25 %)
24–35	43 (54 %)
36–45	24 (30 %)
46–55	7 (8.75 %)
55–65	1 (1.25 %)
Prefer not to answer	4 (5 %)
**Race/ethnicity** ^a^	
American Indian or Alaska Native	3 (3.75 %)
Asian	17 (21.25 %)
Black or African-American	9 (11.25 %)
Hispanic	7 (8.75 %)
White	42 (52.5 %)
Prefer not to answer	3 (2.5 %)
**Gender**	
Cisgender woman	53 (66 %)
Cisgender man	19 (23.75 %)
Nonbinary individual	4 (5 %)
Transgender woman	1 (1.25 %)
Transgender man	3 (3.75 %)
**Sexual orientation**	
Asexual	2 (2.5 %)
Bisexual	11 (13.75 %)
Heterosexual	38 (47.5 %)
Queer/gay/lesbian	29 (36.25 %)
**Household income**	
$0–$30,000	10 (12.5 %)
$31,000–$60,000	4 (5 %)
$61,000–$90,000	20 (25 %)
$91,000–$120,000	4 (5 %)
$120,000+	31 (38.75 %)
Prefer not to answer	11 (13.75 %)

a 14 participants indicated more than one racial/ethnic identity. We list the first category that they listed.

**Table 2:** Progression of focus group scenarios.

Introductions Ice-breakers Brief fertility journey discussions
IVF and infertility statistics Definitions of infertility Types of family formation
IVG science Making reproductive cells from reprogrammed stem cells Biological processes, steps, and challenges
How IVG could work in the clinic in diverse configurations Partners of the same sex or different sexes
Pre-clinical steps to bring IVG to the clinic Attitudes about gamete and embryo creation, research, and disposal Attitudes about volunteering for clinical trials
Hypothetical case scenarios (focus on 2 according to the focus group composition) Different-sex couples (female or male infertility) Gay and lesbian couples Trans and nonbinary individuals and couples
Social Impacts Attitudes about accessibility to IVG Attitudes about family formation Attitudes about IVG’s reception
Key Concerns Attitudes about safety, efficacy, accessibility

## Data Availability

The data that has been used is confidential.
